# Impact of empowering leadership on adaptive performance in hybrid work: a serial mediation effect of knowledge sharing and employee agility

**DOI:** 10.3389/fpsyg.2025.1448820

**Published:** 2025-01-20

**Authors:** Seung-Seok Kim, Dong-Yeol Yoon

**Affiliations:** ^1^Hyundai-steel, Seoul, Republic of Korea; ^2^The Department of Business, Konkuk University, Seoul, Republic of Korea

**Keywords:** empowering leadership, adaptive performance, knowledge sharing, employability, serial mediation effect

## Abstract

With the advent of the pandemic era, many organizations have implemented a hybrid work model to manage office- and remote-based work. The proliferation of hybrid work demands leaders to demonstrate a different style of leadership than that in the past and to delegate authority, providing autonomy and responsibility to employees. Furthermore, both within and outside the organization, members must adapt and respond to rapidly changing environments and demonstrate adaptive performance to generate better outcomes. This study identifies the relationship between empowering leadership and adaptive performance in hybrid work, based on the social exchange and self-determination theories. Furthermore, it validates the mediation effect of knowledge sharing and employee agility and serial mediation effect. We analyzed data from 290 IT manufacturing employees working in hybrid work in South Korea. The findings reveal that empowering leadership positively influences adaptive performance and that knowledge sharing and employee agility partially mediate this relationship. Moreover, knowledge sharing and employee agility serially mediate the impact of empowering leadership on the adaptive performance of employees. These research findings provide theoretical and practical implications for organizations in hybrid work.

## 1 Introduction

Following the COVID-19 pandemic, the economic downturn prompted companies to move beyond traditional work methods and adopt more productive and efficient approaches. Various work methods are encouraged to facilitate the transition from remote work, which has been extensively employed during the pandemic. In this context, hybrid work is expected to emerge as a significant mode of operation in future workplace (Moglia et al., 2021). Specifically, Amazon has informed its employees of plans to reintroduce office-based work to complement the remote work practices established during the pandemic (Amazon, 2023). The company has announced the adoption of a hybrid work model that blends office and remote work arrangements (GeekWire, 2023). This approach aims to leverage the benefits of both work environments, fostering flexibility and maintaining productivity. Thus, hybrid work model is being used to manage remote- and office-based work.

However, compared with traditional work arrangements, the physical distance between leaders and employees increases in hybrid model that reduces communication and feedback, thereby creating a gap in feedback (Zhang et al., 2020). Such physical distance highlights the importance of a different type of leadership compared to traditional settings. In hybrid work arrangements, overcoming the challenges posed by physical separation and achieving strong performance requires employees to take ownership of their tasks and actively engage in their work. Rather than focusing on managing task progress, leaders must guide employees to work autonomously. Therefore, the significance of empowering leadership, which delegates authority to actively engage in their tasks, is growing (Gray et al., 2023; Jolly et al., 2021).

Empowering leadership involves delegating authority to employees, enhancing their intrinsic motivation, and stimulating their sense of responsibility and desire for growth, leading to performance achievements (Zhang and Bartol, 2010). In the current endemic era, it is imperative for organizations and individuals to anticipate, adapt, and respond swiftly to rapid environmental changes. This agility is essential for maintaining resilience and competitiveness in a landscape characterized by ongoing uncertainties and shifting dynamics. By cultivating an adaptive mindset and implementing flexible strategies, organizations can better navigate the complexities of this new era and seize emerging opportunities. Empowering leadership uses distributed authority as a resource to respond agilely to internal and external environmental changes (Arnold et al., 2000).

This study extends the exploration of the mechanisms connecting empowering leadership to adaptive performance by investigating the sequential mediating roles of knowledge sharing and employee agility. Within a knowledge-based society, knowledge is emphasized as a critical resource for enhancing organizational performance and competitiveness (Tung, 2014). Employees actively engage in the exchange and acquisition of knowledge to enhance their capabilities, a process that not only maximizes organizational value but also significantly boosts performance improvement (Srivastava et al., 2006). Under empowering leadership, employees strategically leverage knowledge sharing to enhance performance and respond adeptly to dynamic environments.

Employees must cultivate their skills to effectively adapt to evolving internal and external environmental changes (Kinicki and Latack, 1990). In this context, employee agility has emerged as a crucial competency for navigating dynamic environments. By proactively responding to changes, employees enhance their decision-making capabilities, ultimately leading to improved work quality and performance.

Numerous prior studies have explored the impact of empowering leadership on adaptive performance. However, to achieve a more nuanced understanding of the relationship between these variables, there is a pressing need for integrative research that considers multiple influencing factors (Bonini et al., 2024; Xu and Zhang, 2022). This study aims to investigate the mechanisms through which empowering leadership influences adaptive performance specifically in hybrid work settings, employing self-determination theory and social exchange theory as foundational theoretical frameworks. By examining the mediating roles of knowledge sharing and employee agility within these environments, this study endeavors to deepen the understanding of the connection between empowering leadership and adaptive performance, particularly in the context of increasingly prevalent hybrid work arrangements.

## 2 Literature review

### 2.1 Empowering leadership

Empowering leadership refers to a leadership style in which a leader shares authority with employees, induces intrinsic motivation, and instills them with a sense of the importance of their work and confidence in their performance (Srivastava et al., 2006; Zhang and Bartol, 2010). Traditional leadership faces limitations in responding flexibly to significant changes or resource-scarce environments such as those presented by the COVID-19 pandemic (Javed et al., 2019). In contrast, empowering leadership, by delegating authority and fostering intrinsic motivation, encourages innovative behavior and responds flexibly to changes (Javed et al., 2019).

A leader’s role is vital in fostering employee empowerment (Srivastava et al., 2006; Zhang and Bartol, 2010). Empowerment plays a crucial role in improving individual and organizational performance through self-development and the pursuit of innovative actions. Empowering leadership can be realized from the perspectives of empowerment and leader behavior. The leader behavior perspective focuses on the style of leadership, feedback provision, and behaviors that delegate autonomy and responsibility (Kirkman and Rosen, 1997), focusing on how a leader’s actions impact employees. However, empowerment perspective emphasizes on employees’ responses to delegated authority and motivation, including acquiring and using power and autonomy within the organization (Conger and Kanungo, 1988; Kirkman and Rosen, 1997; Spreitzer, 1995), highlighting how employees perceive and respond to empowering leadership.

Research on empowering leadership examines how leaders’ behavior impacts employees and enhances performance (Druskat and Wheeler, 2003; Judge et al., 2004). According to Zhang and Bartol (2010), empowering leadership positively influences employee performance indicators such as organizational citizenship behavior, innovation, and work performance improvement. Additionally, studies indicate that empowering leadership positively affects employees’ work performance (Kundu et al., 2019).

### 2.2 Adaptive performance

As the Fourth Industrial Revolution is grounded in digitalization, traditional job performance measures have limitations in reflecting the complexity of work (Kim and Yoon, 2021). Moreover, changes in the external environment require employees to enhance their knowledge and skills in response (Kinicki and Latack, 1990). Both the external environment and job roles are becoming more diversified, and the demand for jobs is continuously evolving (Goldstein and Gilliam, 1990). Consequently, the concept of adaptive performance has emerged, which involves actively responding to changing environments that lead to performance outcomes. Adaptive performance refers to appropriately altering one’s responses and actions in new job situations or environments (Pulakos et al., 2000).

Adaptive performance was proposed as a development of the traditional job performance model that distinguishes between task and contextual performance (Allworth and Hesketh, 1999; Ployhart and Bliese, 2006; Pulakos et al., 2000). The increasing complexity and unstructured nature of jobs due to changes in the job environment have prompted the expansion of the concept of job performance. Therefore, individual job performance, previously divided into task and contextual performance, has been expanded to include adaptive performance, reflecting success in adapting to increasing dynamism and changes (Schmitt et al., 2003).

Initially, adaptive performance was defined from a learning perspective (Ployhart and Bliese, 2006). London and Mone (1999) define adaptive performance as a response to new learning, whereas Mumford et al. (1993) define it as the activity of creating new frameworks to adapt to new environments, moving beyond existing learning. However, these definitions are limited, as they focus only on learning behavior and do not fully encompass broader meanings. The scope of adaptive performance is extended to include behaviors required in new environments (Pulakos et al., 2000). This expansion encompasses individual actions based on abilities, dispositions, skills, and proactivity in both the internal and external environments. Furthermore, it has been defined from situational and reactive perspectives, focusing on utilizing various capabilities to improve performance in changing environments (Lepine et al., 2000; Ployhart and Bliese, 2006; Pulakos et al., 2000). Specifically, research has progressed from the perspective that the ability to respond to and proactively utilize capabilities in changing environments leads to performance improvement.

Several studies have explored the multidimensional nature of adaptive performance. Pulakos et al. (2000) distinguish adaptive performance from a situational perspective into eight dimensions: handling emergencies or crisis situations; handling work stress; solving problems creatively; learning work tasks, technologies, and procedures; dealing with uncertain and unpredictable work situations; demonstrating interpersonal adaptability; demonstrating cultural adaptability; and demonstrating physically oriented adaptability. However, Charbonnier-Voirin and Roussel (2012) define it using five dimensions, reactivity in the face of emergencies, creativity, handling work stress, training effort, and interpersonal adaptability, to facilitate generalization.

### 2.3 Knowledge sharing

In a knowledge-based society, knowledge is a crucial resource for sustaining and securing the competitiveness of organizations, with employees voluntarily sharing and learning knowledge playing a key role (Tung, 2014). However, knowledge sharing among individuals and teams must be implemented effectively for knowledge to effectively contribute to gaining a competitive edge (Han et al., 2016). Knowledge sharing includes not only sharing explicit knowledge through documents, manuals, and data but also sharing tacit knowledge such as thoughts, behaviors, and know-how, which can also be communicated through sharing (Nonaka and Takeuchi, 1996). Srivastava et al. (2006) argue that if knowledge is not shared, its value can be underestimated. Therefore, knowledge sharing plays a vital role in realizing the true value of knowledge, beyond merely possessing it.

Van Den Hooff and De Ridder (2004) introduce the concept of knowledge sharing using the concepts of donating and collecting knowledge. Knowledge donation means voluntarily providing the knowledge one possesses to other members, whereas knowledge collection means acquiring knowledge from others through processes such as persuasion (Van Den Hooff and De Ridder, 2004).

A major topic discussed in research related to knowledge sharing is whether a difference exists between the intention to share knowledge and actual knowledge-sharing behavior. According to previous research, intention is a precursor to behavior that transforms into behavior over time (Bandura, 2001). Although the temporal difference between intention and behavior leads them to be perceived as different concepts, the intention to share knowledge influences the behavior leading to actual sharing; hence, it is used as an equivalent concept in the context of knowledge sharing (Reychav and Weisberg, 2010). Therefore, in this study, knowledge sharing is conceptualized and measured according to previous research as the intention to share both explicit and tacit knowledge.

### 2.4 Employee agility

Owing to the unpredictability of recent environments, the importance of employees’ agile mindsets has been emphasized (Harsch and Festing, 2019). Additionally, in the era of digital transformation, agility enables employees to quickly adapt and respond to change, allowing the execution of appropriate strategies (Nijssen and Paauwe, 2012; Troise et al., 2022; Baran and Woznyj, 2020). Organizations with agile employees can respond flexibly to environmental changes and secure a competitive advantage.

The concept of agility has been studied from both personal and organizational perspectives (Breu et al., 2002; Salmen and Festing, 2022). Organizational agility is defined as the ability to anticipate and leverage changes in the external environment to respond quickly (Sharifi and Zhang, 1999; Sherehiy and Karwowski, 2014). According to prior research, organizational agility largely depends on individual agility (Harsch and Festing, 2019). Individual agility must be improved to respond to changes at the organizational level. Therefore, this study validates the concept of employee agility at an individual level.

Early research defines agile employees as those who effectively solve given problems and pursue self-development (Plonka, 1997). Later, Breu et al. (2010) define it in terms of technical aspects such as a combination of skills, responsiveness to change, and access to information. From a behavioral perspective, it is defined as the skill to anticipate changes, learning new technologies, and responding flexibly (Sherehiy and Karwowski, 2014). Braun et al. (2017) integrate these concepts to define employee agility as a skill to actively respond to changes. They divide employee agility into three subfactors according to work adjustment theory: proactivity, adaptivity, and resilience. Proactivity refers to the active response to problems effectuated by changes in anticipation of solving them. Adaptivity refers to the voluntary learning and acquisition of skills to adapt to environmental changes. Finally, resilience refers to the efficient management of the unpredictable stress caused by changes. Owing to the ambiguity regarding the concept of resilience, recent research has emphasized the importance of proactivity and adaptivity, which involve predicting new environments and acting proactively (Pitafi et al., 2018).

## 3 Hypothesis development

### 3.1 Empowering leadership and adaptive performance

Empowering leaders enhance employees’ intrinsic motivation (Chen et al., 2011; Lorinkova and Perry, 2017; Zhang and Bartol, 2010). When delegating authority, employees voluntarily participate in decision-making processes and actively express their opinions. This process makes them recognize the importance of their work and the organization’s goals, leading to intrinsic motivation (Amundsen and Martinsen, 2014). Thus, enhanced intrinsic motivation is utilized as a resource in changing environments, influencing the improvement of adaptive performance.

According to the self-determination theory (Ryan and Deci, 2000), to enhance intrinsic motivation for performance, psychological needs such as competence, autonomy, and relatedness must be satisfied. Empowering leadership can fulfill these psychological needs by delegating authority. Delegated authority increases autonomy in job roles and strengthens relationships with employees through trust (Morrison and Phelps, 1999; Zhang et al., 2020). Thus, enhanced intrinsic motivation is utilized as a resource to respond to changes and challenging tasks (Bajaba et al., 2021; Griffin et al., 2010; Niessen and Jimmieson, 2016). Specifically, enhanced intrinsic motivation makes individuals feel responsible for autonomous decisions, preparing them to face the risks of change and strive to improve their performance (Demerouti and Bakker, 2011).

This relationship can be explained by the social exchange theory (Blau, 1964; Porter, 2018). According to the social exchange theory, individuals reciprocate favors they receive, adhering to the norm of reciprocity. Employees perceive delegated authority as a sign of trust and favor and seek to reciprocate it. In an effort to reciprocate, they maintain a positive attitude and engage in behaviors beneficial to the organization. Such constructive behavior is utilized as a resource that positively influences adaptive performance.

In hybrid work environments, the physical distance between leaders and employees frequently results in feedback gaps (Zhang et al., 2020). These gaps hinder leaders’ ability to promptly intervene in employees’ tasks, necessitating that employees independently reflect on, adjust, and execute their work to achieve desired outcomes. In such contexts, it is imperative that employees work autonomously and approach their tasks with intrinsic motivation. Empowering leadership plays a crucial role by fostering autonomy and responsibility, encouraging employees to take ownership of their work. This leadership approach fulfills essential psychological needs, thereby enhancing intrinsic motivation and positively influencing adaptive performance.

*H1*: Empowering leadership positively affects adaptive performance in hybrid work.

### 3.2 Mediation effect of knowledge sharing

In the digital era, knowledge becomes increasingly crucial. However, even the most valuable knowledge has limited potential to enhance organizational performance and individual competencies if not shared or used by employees (Nonaka, 1991). Therefore, to fully leverage knowledge as a competitive advantage for an organization, it is essential to consider how employees share the knowledge they possess (Argote, 2012).

As knowledge acts as a key competitive factor in this era, empowering leadership plays a critical role in fostering individual autonomy and affecting knowledge sharing (Singh, 2008). Employees endowed with authority and responsibility over their work require appropriate knowledge and information for rational decision-making. Hence, they strive to secure resources for decision-making by sharing knowledge, information, and know-how. Consequently, empowering leadership drives employees’ knowledge sharing using it as a resource impacting adaptive performance (Oliver and Reddy Kandadi, 2006; Srivastava et al., 2006).

The mediation effect of knowledge sharing can be explained by the self-determination theory (Ryan and Deci, 2000). As voluntary knowledge sharing is nonmandatory, motivation is necessary (Peng, 2013; Wang and Noe, 2010). Enhanced intrinsic motivation through empowering leadership encourages individuals to determine and improve the resources required to better perform their tasks (Ahearne et al., 2005). Through such efforts, shared knowledge becomes a resource that is utilized to adapt to changing and unfamiliar environments, thus contributing to performance.

This relationship can also be explained by the social exchange theory (Blau, 1964; Porter, 2018). Employees empowered through empowering leadership engage in constructive actions for the organization (Arnold et al., 2000; Rao Jada et al., 2019). As part of these efforts, employees willingly share their intellectual assets such as information, knowledge, and know-how with others and actively seek knowledge to achieve better performance. In other words, to reciprocate the favor of the leader, they share their intellectual assets and competitiveness for the growth and development of the organization, despite the potential risk of losing their competitive edge. These intellectual resources are used to proactively respond to changes and achieve performance outcomes.

In hybrid work environments, the physical separation from colleagues often leads to reduced access to resources compared to traditional office settings. To better adapt to changes and achieve higher performance under these conditions, employees must proactively gather resources. Specifically, they are driven to engage in knowledge sharing to acquire and expand their intellectual capital, enabling them to perform their assigned tasks more effectively. This acquired knowledge enhances decision-making quality and acts as a critical resource for responding to and adapting to environmental changes, thereby positively influencing adaptive performance.

*H2*: Knowledge sharing mediates the relationship between empowering leadership and adaptive performance in hybrid work.

### 3.3 Mediation effect of employee agility

Rapid environmental changes require employees to develop the appropriate skills (Kinicki and Latack, 1990). In response to such changes, the significance of skills that prepare employees to proactively adapt to changing environments based on resources and knowledge, known as employee agility, has been emphasized.

Employee agility and adaptive performance share conceptual similarities in their responses to changes. However, employee agility is defined as the skill to respond to change, whereas adaptive performance is defined as the behavior of responding and adapting to actual changes or unfamiliar environments.

Furthermore, the mediating effect of employee agility is explained by the self-determination theory (Ryan and Deci, 2000). Authority and autonomy achieved through empowering leadership serve as motivations for better decision-making. According to the self-determination theory, employees whose psychological needs are satisfied voluntarily strive to develop skills (Ahearne et al., 2005). They seek, train, and prepare for the skills required to respond appropriately to internal and external organizational environments. These efforts positively impact employee agility. The skill of anticipating and responding to change assists in efficiently reacting and adapting to actual changes and unfamiliar environments, thus contributing to performance outcomes.

According to the social exchange theory (Blau, 1964; Porter, 2018), the favor perceived by employees owing to empowering leadership induces them to engage in constructive behaviors to reciprocate with the organization and leader. Employees contribute to an organization by enhancing skills that predict and respond to unpredictable changes, managing future challenges, and handling organizational risks. These skills aid in adapting and responding to actual changes and unfamiliar environments, thereby contributing to performance outcomes.

In a hybrid work setting, employees face unpredictable changes in both office and remote work environments. Moreover, working in new locations and adopting new work styles introduce additional changes. In such environments, employees with intrinsic motivation fostered by empowering leadership endeavor to develop skills to predict and manage change. Enhanced skills can be applied to proactively respond and adapt to unpredictable changes occurring in a hybrid work setting, contributing to performance improvement.

*H3*: Employee Agility mediates the relationship between empowering leadership and adaptive performance in hybrid work.

### 3.4 Serial mediation effect of knowledge sharing and employee agility

According to the self-determination theory (Ryan and Deci, 2000), employees who receive delegated authority through empowering leadership experience satisfaction with their psychological needs, enhancing their intrinsic motivation. This enhancement enables employees to select and utilize the resources necessary for better task performance, thereby improving decision-making quality (Ahearne et al., 2005). Employees actively participate in knowledge sharing to acquire such resources. These intellectual assets enhance skills for predicting and responding to internal and external organizational changes. These efforts are utilized as resources to appropriately respond to actual changes, thereby impacting adaptive performance.

This perspective can also be affirmed through the social exchange theory (Blau, 1964; Porter, 2018). Leaders’ behaviors, such as those exhibited in empowering leadership, act as positive experiences for employees and motivate them to reciprocate. Employees voluntarily share their knowledge and strive to learn from others for personal and organizational growth and performance. They not only apply the acquired intellectual assets to their tasks but also use them to enhance their skills to predict and respond to potential changes. These risk management skills are applied when actual changes occur, managing organizational change and risks as reciprocating behaviors toward the organization.

**Figure 1:**
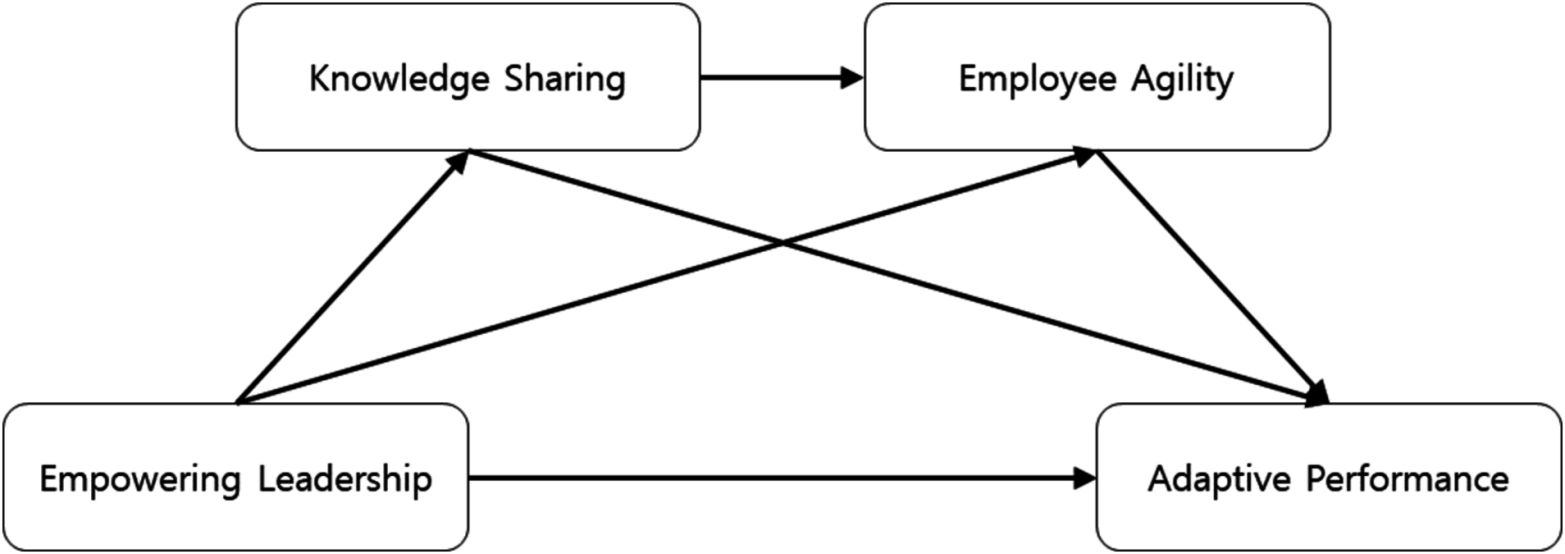
Research model.

In a hybrid work environment, where access to resources may be more limited than in traditional work settings, employees with delegated authority actively strive to acquire intellectual assets for better decision-making. Moreover, they utilize these intellectual assets to enhance skills for timely response to changes that may occur in the new work environment, facilitating better task performance. Such actions serve as resources for timely response and adaptation to the unpredictable changes attributed to hybrid work, thereby impacting adaptive performance (Figure 1).

*H4*: Empowering leadership influences knowledge sharing, affecting employee agility and subsequently adaptive performance in hybrid work. Specifically, the relationship between empowering leadership and adaptive performance is serially mediated by knowledge sharing and employee agility in hybrid work.

## 4 Research methodology

### 4.1 Sample and data collection

We collected data through both online and offline surveys targeting employees from small- and medium-sized IT manufacturing companies in South Korea who were engaged in hybrid work during the COVID-19 pandemic. This study specifically focused on IT-based firms utilizing hybrid work models, with an emphasis on the IT manufacturing sector, where adaptive performance is considered particularly crucial for effectively responding to changes. The surveys were administered exclusively to employees actively participating in hybrid work arrangements from March to April 2022. After removing insincere responses and incomplete data, a total of 290 valid responses were obtained and analyzed, comprising 258 online and 32 offline submissions (see Table 1 for details).

**Table 1:** Frequency analysis results.

Classification		Frequency	Percentage
Gender	Male	197	67.9
	Female	93	32.1
Age	20–29 years old	42	14.5
	30–39 years old	125	43.1
	40–49 years old	65	22.4
	More than 50 years old	58	20.0
Education	High school	11	3.8
	College	29	10.0
	Undergraduate	173	59.6
	Master’s degree	62	21.4
	Doctorate degree	15	5.2
Job	R&D	105	36.2
	Management	88	30.3
	Other	97	33.5
Position	Staff	53	18.3
	Associate	15	5.2
	Senior associate	42	14.5
	Manager	54	18.6
	Senior manager	32	11.0
	Executive manager	38	13.1
	Executive	56	19.3
Job tenure	Less than 2 years	56	19.3
	2–5 years	50	17.2
	5–8 years	47	16.2
	8–10 years	33	11.4
	10–13 years	25	8.6
	More than 13 years	79	27.2

### 4.2 Data analysis method

SPSS 26.0 and AMOS 23.0 softwares were used to analyze the data. Confirmatory factor analysis (CFA) was conducted to assess the discriminant, convergent, and conceptual validity of the measurement variables. Subsequently, correlation analysis was performed to examine the relationships between the measurement variables. Finally, we employed the bootstrap method (Hayes, 2013) in the PROCESS macro to test the serial mediation model. We used Model 6 of the PROCESS macro to validate the serial mediation model.

### 4.3 Variable measurement

#### 4.3.1 Empowering leadership

Empowering leadership refers to the behavior of leaders who share authority with employees, thereby increasing their intrinsic motivation and providing them with confidence in the importance and performance of their work (Srivastava et al., 2006; Zhang and Bartol, 2010). The measurement tool for empowering leadership utilized a 5-point scale with 12 items from Ahearne et al. (2005). It comprised sub-dimensions such as enhancing the meaningfulness of work (three items), fostering participation in decision-making (three items), expressing confidence in high performance (three items), and providing autonomy from bureaucratic constraints (three items).

#### 4.3.2 Adaptive performance

Adaptive performance is defined as appropriately changing one’s behavior and responses to align with the demands of new situations or job environments (Pulakos et al., 2000). It was measured using a 5-point scale with 19 items developed by Charbonnier-Voirin and Roussel (2012). The subdimensions of adaptive performance included reactivity during emergencies (four items), handling work stress (three items), creativity (four items), training effort (four items), and interpersonal adaptability (four items).

#### 4.3.3 Knowledge sharing

Knowledge sharing is the intention to share explicit and implicit knowledge (Bock et al., 2005). Following previous research, knowledge-sharing intentions and knowledge-sharing behaviors were conceptualized and used as the same terms (Reychav and Weisberg, 2010). It was measured using a 5-point scale with five items developed by Bock et al. (2005). The items included intention to share explicit knowledge (two items) and intention to share implicit knowledge (three items).

#### 4.3.4 Employee agility

Employee agility is the skill of employees to proactively utilize their knowledge and skills to adapt to new opportunities and environments (Braun et al., 2017). This was measured using a 5-point scale with five items developed by Braun et al. (2017). In this study, four items were utilized for the analysis, excluding one item that inquired about past experiences of responding to actual changes rather than the proactive behavior of employee agility. Additionally, based on the results of the CFA, this particular item did not adequately explain the concept, with a coefficient of 0.575.

#### 4.3.5 Control variables

Gender, age, education level, job category, position, and tenure were considered control variables. Gender and job category were dummy coded as R&D and Management, respectively. Regarding gender, females were coded as 0 and males as 1. The two most common job categories, R&D and Management, were selected. R&D was coded as 1, and other job categories were coded as 0 for the analysis.

## 5 Empirical results

### 5.1 Reliability analysis of variables

A reliability analysis was conducted to verify the validity of the measurement instruments. The reliability verification uses Cronbach’s *α* coefficient ranging from 0 to 1, and a value of 0.6 or higher indicates high reliability (Morgan et al., 2004). In this study, empowering leadership indicates a value of 0.927, adaptive performance 0.930, knowledge sharing 0.918, and employee agility 0.878. As all variables exceed 0.6, they are considered to demonstrate high reliability.

### 5.2 Confirmatory factor analysis

The results of the CFA indicate that the average variance extracted (AVE) values for all variables are above 0.5: empowering leadership (0.744), adaptive performance (0.682), knowledge sharing (0.692), and employee agility (0.582). The values for construct reliability (CR) are empowering leadership (0.972), adaptive performance (0.975), knowledge sharing (0.918), and employee agility (0.872), all of which exceeded 0.7. According to Anderson and Gerbing (1988) and Fornell and Larcker (1981), a model is considered appropriate when AVE is above 0.5 and CR is above 0.7.

The overall model fit is also assessed using CFA, yielding a chi-square of 1412.618, root mean square residual of 0.051, root mean square error of approximation of 0.055, and a comparative fit index of 0.917. Previous research suggests that an RMSEA value below 0.08 and a CFI value above 0.9 indicate a good fit (Bentler, 1990; MacCallum et al., 1996). Therefore, this model is considered appropriate.

### 5.3 Correlation analysis

We conducted Pearson’s correlation analysis to examine the relationships among variables, and the results are presented in Table 2. The findings reveal that Empowering leadership has a significant positive correlation with knowledge sharing (*r* = 0.476, *p* < 0.001), employee agility (*r* = 0.384, *p* < 0.001), and adaptive performance (*r* = 0.478, *p* < 0.001). Furthermore, knowledge sharing shows a significant positive correlation with employee agility (*r* = 0.341, *p* < 0.001) and adaptive performance (*r* = 0.567, *p* < 0.001), whereas employee agility has a significant positive correlation with adaptive performance (*r* = 0.687, *p* < 0.001).

**Table 2:** Descriptive statistics and correlation results.

	Mean	SD	1	2	3	4	5	6	7	8	9	10	11
1. Gender	0.68	0.46	–										
2. Age	2.48	0.97	−0.256^***^	–									
3. Education	4.14	0.80	−0.065	0.345^***^	–								
4. Management	0.30	0.46	0.061	0.239^***^	0.117^*^	–							
5. R&D	0.36	0.48	−0.026	−0.254^***^	−0.096	−0.497^***^	–						
6. Position	4.16	2.08	−0.221^***^	0.825^***^	0.415^***^	0.250^***^	−0.259	–					
7. Tenure	3.54	1.89	−0.210^***^	0.636^***^	0.262^***^	0.135^*^	−0.191^**^	0.663^***^	–				
8. Empowering leadership	3.57	0.66	−0.036	−0.021	0.026	0.047	0.140^*^	0.019	−0.125^*^	–			
9. Knowledge sharing	3.99	0.69	−0.035	0.082	0.092	−0.025	0.076	0.145^*^	−0.006	0.476^***^	–		
10. Employee agility	3.39	0.77	−0.095	0.266^***^	0.184^**^	0.235^***^	−0.172^**^	0.307^***^	0.209^***^	0.384^***^	0.341^***^	–	
11. Adaptive performance	3.62	0.54	−0.074	0.312^***^	0.210^***^	0.071	−0.043	0.303^***^	0.120^*^	0.478^***^	0.567^***^	0.687^***^	–

*n* = 290, *p < 0.05, **p < 0.01, ***p < 0.001.

1. Age (dummy variable): 0 = Women, 1 = Men, 2. Age: 1 = Twenties, 2 = Thirties, 3 = Forties, 4 = Over fifties, 3. Education: 1 = Less than high school graduate, 2 = High school graduate, 3 = Technical college graduate, 4 = University graduate, 5 = Master, 6 = Doctor, 4. Job Group (dummy variable): 1 = Management support, 0 = Production/purchase, R&D, sales/marketing/service, etc., 5. Job group (dummy variable): 1 = R&D, 0 = Management support, production/purchase, sales/marketing/service, etc., 6.Position: 1 = staff, 2 = Administrative manager, 3 = Assistant manager, 4 = Manager, 5 = Deputy general manager, 6 = Department manager, 7 = Executive officer, 7. Tenure: 1 = Less than 2 years, 2 = 2 ~ 5 years, 3 = 5 ~ 8 years, 4 = 8 ~ 10 years, 5 = 10 ~ 13 years, 6 = 13 years or more.

### 5.4 Hypothesis testing and results

We hypothesized that knowledge sharing and employee agility indirectly affect the direct relationship between empowering leadership and adaptive performance. To test this hypothesis, we utilized Baron and Kenny (1986)’s three-step mediation analysis and Hayes (2013) serial mediation effect model analysis using the SPSS Process Macro Model 6. All analyses were conducted using 10,000 bootstrap resamples to obtain 95% confidence intervals (CI).

To verify this hypothesis, we conducted a hierarchical regression analysis, and the results are presented in Tables 3, 4. Model 6 in Table 4 shows that empowering leadership has a significant effect on adaptive performance (*β* = 0.483, *p* < 0.001), indicating statistical significance (∆R^2 = 0.216, F for ∆R^2 = 93.211, *p* < 0.001). This result supports Hypothesis 1.

**Table 3:** Regression results.

Variable	Knowledge sharing		Employee agility	
	Model 1	Model 2	Model 3	Model 4
Gender	−0.008	0.016	−0.051	−0.030
Age	−0.058	−0.034	0.004	0.025
Education	0.035	0.028	0.068	0.062
Management	−0.019	−0.085	0.161^*^	0.104
R&D	0.106	0.008	−0.029	−0.144
Position	0.322^**^	0.222^*^	0.209	0.123
Tenure	−0.170^*^	−0.064	0.012	0.103
Empowering Leadership	–	0.466^***^	–	0.403^***^
*R* ^2^	0.055	0.257	0.128	0.279
adj.	0.031	0.235	0.106	0.259
*∆R* ^2^	–	0.202	–	0.151
F for	2.342^*^	76.220^***^	5.917^***^	58.872^***^
Overall F	2.342^*^	12.123^***^	5.917^***^	13.599^***^

*n* = 290, *p < 0.05, **p < 0.01, ***p < 0.001; standardized beta.

**Table 4:** Regression results.

Variable	Adaptive performance			
	Model 5	Model 6	Model 7	Model 8
Gender	0.001	0.026	0.020	0.043
Age	0.248^*^	0.274^**^	0.287	0.259^***^
Education	0.097	0.090	0.079	0.055
Management	−0.001	−0.070	−0.035	−0.128^**^
R&D	0.044	−0.057	−0.060	0.007
Position	0.189	0.085	−0.003	0.016
Tenure	−0.180^*^	−0.070	−0.044	−0.129^*^
Empowering leadership		0.483^***^	0.297^***^	0.254^***^
Knowledge sharing			0.400^***^	
Employee agility				0.568^***^
*R* ^2^	0.131	0.348	0.466	0.580
adj.	0.110	0.329	0.449	0.567
*∆R* ^2^	–	0.216	0.119	0.232
F for	6.097^***^	93.211^***^	62.297^***^	154.913^***^
Overall F	6.097^***^	18.731^***^	27.204^***^	42.982^***^

*n* = 290, *p < 0.05, **p < 0.01, ***p < 0.001; standardized beta.

Baron and Kenny’s (1986) three-step mediation analysis is used to verify the mediating effects of knowledge sharing and employee agility. Models 2 and 4 in Table 3 show that empowering leadership has a direct positive impact on the mediating variables, knowledge sharing, and employee agility. Furthermore, Model 6 in Table 4 illustrates, as confirmed by Hypothesis 1, that empowering leadership significantly affects adaptive performance. Model 7 in Table 4 demonstrates that both empowering leadership and knowledge sharing significantly impact adaptive performance (*β* = 0.297, *p* < 0.001; *β* = 0.400, *p* < 0.001), with statistical significance (∆R^2 = 0.119, F for ∆R^2 = 62.297, *p* < 0.001). Similarly, the analysis regarding employee agility also indicates a significant effect (*β* = 0.254, *p* < 0.001; *β* = 0.568, p < 0.001), with statistical significance (∆R^2 = 0.232, F for ∆R^2 = 154.913, *p* < 0.001).

Following Baron and Kenny (1986)’s criteria for a partial mediation effect, both variables exhibit a partial mediation effect. Additionally, a more precise Sobel test shows significant mediating effects, with Z-values of 5.8484 for knowledge sharing and 6.5320 for employee agility as mediators. In the Sobel test, a Z-value greater than 1.96 or less than −1.96 indicates a significant mediating effect (Baron and Kenny, 1986). These results support Hypotheses 2 and 3 (Table 5).

**Table 5:** Effect of empowering leadership on adaptive performance.

Total effect of empowering leadership on adaptive performance					
*B*	SE	*t*	*p*	LLCI	ULCI
0.3998	0.0414	9.6546	0.0000	0.3183	0.4814

Direct effect of empowering leadership on adaptive performance					
*B*	SE	*t*	*p*	LLCI	ULCI
0.1106	0.0362	3.0548	0.0025	0.0393	0.1819

Boot LLCI: Lower limit within the 95% confidence interval of the bootstrap indirect effect.

Boot ULCI: Upper limit within 95% confidence interval of Bootstrap indirect effect.

To examine the serial mediation effect of knowledge sharing and employee agility, Hayes (2017) Process Macro Model 6 was used. Table 6 illustrates the serial mediation effects of knowledge sharing and employee agility on the relationship between empowering leadership and adaptive performance. The magnitude of the effect is 0.0356, with a 95% confidence interval (LLCI = 0.0099, ULCI = 0.0638), indicating significance. Furthermore, the size of the indirect effect for the entire model presented in Table 6 is 0.2892 (LLCI = 0.2205, ULCI = 0.3596), showing significance at the 95% confidence level. These results support Hypothesis 4.

**Table 6:** Serial mediation effect results.

Indirect effect of empowering leadership on adaptive performance				
	*B*	SE	LLCI	ULCI
Total	0.2892	0.0353	0.2205	0.3596
EL – KS – AP	0.1187	0.0199	0.0818	0.1596
EL – AG – AP	0.1350	0.0288	0.0800	0.1924
EL – KS – AG - AP	0.0356	0.0137	0.0099	0.0638

Boot LLCI: Lower limit within the 95% confidence interval of the bootstrap indirect effect.

Boot ULCI: Upper limit within 95% confidence interval of Bootstrap indirect effect.

## 6 Conclusion and future research

### 6.1 Summary and theoretical implications

This study provides a comprehensive empirical examination of the mechanisms through which empowering leadership influences adaptive performance in hybrid work environments. Unlike traditional settings, hybrid work introduces physical distances between leaders and employees, as well as among the employees themselves. Understanding how to bridge these distances to enhance adaptive performance necessitates an exploration of the role and mechanisms of empowering leadership. Our findings reveal that the influence of empowering leadership on adaptive performance extends beyond conventional work contexts, being equally significant within hybrid work settings.

Moreover, this research investigates the mediating effects of knowledge sharing and employee agility on the relationship between empowering leadership and adaptive performance. To fully elucidate this relationship, identifying the mediating factors is essential. The study validates that the delegation of authority through empowering leadership enhances intrinsic motivation, which in turn fosters knowledge sharing and employee agility, ultimately boosting adaptive performance.

Lastly, the research highlights the serial mediating effect of knowledge sharing and employee agility. Expanding the understanding of the mechanisms linking empowering leadership to adaptive performance requires an integrative examination of the sequential relationships among various variables (Bonini et al., 2024). By confirming this serial mediating effect, the study contributes to a more profound understanding of the underlying processes, thereby enriching the theoretical and practical insights into adaptive performance in hybrid work environments.

### 6.2 Practical implications

This study underscores the vital role of empowering leadership in enhancing employee adaptability and responsiveness to unpredictable changes within hybrid work environments. As organizations shift from strictly remote to office-based operations, they are increasingly adopting hybrid work models as a sustainable alternative. However, these settings pose challenges to traditional management approaches that focus on closely overseeing every aspect of team activities. Empowering leadership, characterized by the delegation of authority, grants employees the autonomy and responsibility needed to perform tasks independently. This autonomy fosters intrinsic motivation, which drives employees to effectively adapt to changing environments and achieve improved performance. Consequently, organizations operating in hybrid settings can enhance adaptive performance by training leaders on the significance and practice of delegation. Additionally, minimizing unnecessary reporting requirements and alleviating decision-making bottlenecks can indirectly promote empowering leadership by enabling employees to take greater ownership of their work. To directly promote empowering leadership in a hybrid work environment, organizations can implement leadership training programs. These programs should educate leaders on the differences between office-based and hybrid work environments and help them understand how employees perform their tasks in such settings. Additionally, coaching or group training sessions can be conducted to guide leaders on effective communication strategies for delegating authority. For empowering leadership to be effectively practiced, communication and relationships between leaders and employees are crucial. To genuinely delegate authority, organizations must refine their work and communication processes, enabling leaders to better manage these aspects. By developing these competencies, leaders can positively influence adaptive performance within the organization.

Furthermore, the study reveals that empowering leadership fosters knowledge sharing and employee agility, both of which are crucial for influencing adaptive performance in hybrid work environments. The physical separation inherent in hybrid work can impede the flow of explicit and tacit knowledge, essential for maintaining organizational competitiveness. In this context, emphasizing empowering leadership can enhance the exchange and dissemination of knowledge among employees. Moreover, empowering leadership bolsters employee agility, a critical capability for navigating rapidly changing environments, particularly those experiencing advancements in AI and technology. By delegating appropriate authority, empowering leadership enables employees to become more agile and, consequently, achieve superior adaptive performance. Therefore, improving adaptive performance necessitates not only training leaders in empowering leadership but also establishing systems and creating environments that encourage knowledge sharing and enhance employee agility. These integrated strategies collectively contribute to improved organizational adaptability and competitiveness in dynamic and uncertain environments.

### 6.3 Limitations and future research directions

First, it focuses on the IT manufacturing industry, which faces many changes, where intellectual assets are relatively more important. However, these effects may vary depending on the industry and job roles. Therefore, future research should analyze the operation of mechanisms in general situations, considering industry and job roles.

Second, this study focuses on the positive effects of empowering leadership in a hybrid work environment. However, employees may perceive excessive delegation of authority as an avoidance of responsibility. According to previous research, there is a curvilinear relationship between empowering leadership and workplace performance (Lee et al., 2017). In addition, it may have negative effects depending on the specific job and characteristics of the employee (Wang and Sun, 2019). Therefore, future research should empirically examine the negative aspects of empowering leadership and analyze its impact on adaptive performance under various conditions.

Third, although multicollinearity between employee agility and adaptive performance was not observed in this study, caution should be exercised because of the similarity of the constructs. In this study, employee agility is defined as skills developed before a change occurs, and adaptive performance is defined as behaviors that appropriately respond and adapt when actual changes occur. Future research should conduct a deeper review of the two variables to analyze the differences explicitly.

Fourth, this study used a cross-sectional design that involved surveys at a specific point in time. To analyze the causal relationship between empowering leadership and adaptive performance explicitly, it is necessary to analyze these variables at different time frames. Future research should use longitudinal studies to empirically analyze the causal relationships between mechanisms.

Fifth, this study focused on the positive effects of empowering leadership. However, in the complex dynamics of work types, methods, and relationships between leaders and members, empowering leadership may also have negative impacts. Future research should explore both the positive and negative effects of empowering leadership, striving to achieve a balanced understanding.

Finally, we used the self-reporting method, which can cause common method bias (Podsakoff et al., 2003). Future research should verify more accurate mechanisms using evaluations from supervisors and colleagues, actual performance data, along with self-reporting.

## Data Availability

The raw data supporting the conclusions of this article will be made available by the authors, without undue reservation.

## References

[ref1] Ahearne M. Mathieu J. Rapp A. (2005). To empower or not to empower your sales force? An empirical examination of the influence of leadership empowerment behavior on customer satisfaction and performance. J. Appl. Psychol. 90, 945–955. doi: 10.1037/0021-9010.90.5.945 16162066

[ref2] Allworth E. Hesketh B. (1999). Construct-oriented biodata: capturing change-related and contextually relevant future performance. Int. J. Sel. Assess. 7, 97–111.

[ref3] Amazon. (2023). Update from Andy Jassy on return to office plans. Available at: https://www.aboutamazon.com/news/company-news/andy-jassy-update-on-amazon-return-to-office.

[ref4] Amundsen S. Martinsen Ø. L. (2014). Empowering leadership: construct clarification, conceptualization, and validation of a new scale. Leadersh. Q. 25, 487–511. doi: 10.1016/j.leaqua.2013.11.009

[ref5] Anderson J. C. Gerbing D. W. (1988). Structural equation modeling in practice: a review and recommended two-step approach. Psychol. Bull. 103, 411–423. doi: 10.1037/0033-2909.103.3.411

[ref6] Argote L. (2012). Organizational learning: Creating, retaining and transferring knowledge. Berlin: Springer Science & Business Media.

[ref7] Arnold J. A. Arad S. Rhoades J. A. Drasgow F. (2000). The empowering leadership questionnaire: the construction and validation of a new scale for measuring leader behaviors. J. Organ. Behav. 21, 249–269. doi: 10.1002/(SICI)1099-1379(200005)21:3<249::AID-JOB10>3.0.CO;2-#

[ref8] Bajaba A. Bajaba S. Algarni M. Basahal A. Basahel S. (2021). Adaptive managers as emerging leaders during the COVID-19 crisis. Front. Psychol. 12:661628. doi: 10.3389/fpsyg.2021.661628 33927672 PMC8076558

[ref9] Bandura A. (2001). Social cognitive theory: an agentic perspective. Annu. Rev. Psychol. 52, 1–26. doi: 10.1146/annurev.psych.52.1.1 11148297

[ref10] Baran B. E. Woznyj H. M. (2020). Managing VUCA: The human dynamics of agility. Managing VUCA: Organizational Dynamics. doi: 10.1016/j.orgdyn.2020.100787 PMC7439966 32843777

[ref11] Baron R. M. Kenny D. A. (1986). The moderator–mediator variable distinction in social psychological research: conceptual, strategic, and statistical considerations. J. Pers. Soc. Psychol. 51, 1173–1182. doi: 10.1037/0022-3514.51.6.1173 3806354

[ref12] Bentler P. M. (1990). Comparative fit indexes in structural models. Psychol. Bull. 107, 238–246. doi: 10.1037/0033-2909.107.2.238 2320703

[ref13] Blau P. M. (1964). Justice in social exchange. Sociol. Inquiry 34, 193–206. doi: 10.1111/j.1475-682X.1964.tb00583.x

[ref14] Bock G. W. Zmud R. W. Kim Y. G. Lee J. N. (2005). Behavioral intention formation in knowledge sharing: examining the roles of extrinsic motivators, social-psychological forces, and organizational climate. MIS Q. 29, 87–111. doi: 10.2307/25148669

[ref15] Bonini A. Panari C. Caricati L. Mariani M. G. (2024). The relationship between leadership and adaptive performance: a systematic review and meta-analysis. PLoS One 19:e0304720. doi: 10.1371/journal.pone.0304720 39423211 PMC11488715

[ref16] Braun T. J. Hayes B. C. DeMuth R. L. F. Taran O. A. (2017). The development, validation, and practical application of an employee agility and resilience measure to facilitate organizational change. Ind. Organ. Psychol. 10, 703–723. doi: 10.1017/iop.2017.79

[ref17] Breu K. Hemingway C. J. Strathern M. Bridger D. (2002). Workforce agility: the new employee strategy for the knowledge economy. J. Inf. Technol. 17, 21–31. doi: 10.1080/02683960110132070

[ref18] Breu S. Premraj R. Sillito J. Zimmermann T. (2010). Information needs in bug reports: improving cooperation between developers and users. In Proceedings of the 2010 ACM conference on computer supported cooperative work (pp. 301–310).

[ref19] Charbonnier-Voirin A. Roussel P. (2012). Adaptive performance: a new scale to measure individual performance in organizations. Canad. J. Admin. Sci. 29, 280–293. doi: 10.1002/cjas.232

[ref20] Chen G. Sharma P. N. Edinger S. K. Shapiro D. L. Farh J. L. (2011). Motivating and demotivating forces in teams: cross-level influences of empowering leadership and relationship conflict. J. Appl. Psychol. 96, 541–557. doi: 10.1037/a0021886 21171730

[ref21] Conger J. A. Kanungo R. N. (1988). The empowerment process: integrating theory and practice. Acad. Manag. Rev. 13, 471–482. doi: 10.2307/258093

[ref23] Demerouti E. Bakker A. B. (2011). The job demands-resources model: challenges for future research. SA J. Ind. Psychol. 37, 01–09. doi: 10.4102/sajip.v37i2.974

[ref24] Druskat V. Wheeler J. (2003). Managing from the boundary: the effective leadership of self-managing work teams. Acad. Manag. J. 46, 435–457. doi: 10.2307/30040637

[ref25] Fornell C. Larcker D. F. (1981). Evaluating structural equation models with unobservable variables and measurement error. J. Mark. Res. 18, 39–50. doi: 10.1177/002224378101800104

[ref26] GeekWire. (2023). Amazon changes back-to-office policy, tells corporate workers to come in 3 days a week. Available at: https://www.geekwire.com/2023/amazon-changes-back-to-office-policy-tells-corporate-workers-to-come-in-3-days-a-week.

[ref27] Goldstein I. L. Gilliam P. (1990). Training system issues in the year 2000. Am. Psychol. 45, 134–143. doi: 10.1037/0003-066X.45.2.134

[ref28] Gray C. E. Spector P. E. Wells J. E. Bianchi S. R. Ocana-Dominguez C. Stringer C.. (2023). How can organizational leaders help? Examining the effectiveness of leaders’ support during a crisis. J. Bus. Psychol. 38, 215–237. doi: 10.1007/s10869-022-09810-6 35431433 PMC8995167

[ref29] Griffin M. A. Parker S. K. Mason C. M. (2010). Leader vision and the development of adaptive and proactive performance: a longitudinal study. J. Appl. Psychol. 95, 174–182. doi: 10.1037/a0017263 20085414

[ref30] Han S. H. Seo G. Yoon S. W. Yoon D. Y. (2016). Transformational leadership and knowledge sharing. J. Work. Learn. 28, 130–149. doi: 10.1108/JWL-09-2015-0066

[ref31] Harsch K. Festing M. (2019). Dynamic talent management capabilities and organizational agility—a qualitative exploration. Hum. Resour. Manag. 59, 43–61. doi: 10.1002/hrm.21972

[ref32] Hayes A. F. (2013). Mediation, moderation, and conditional process analysis. Introduction to mediation, moderation, and conditional process analysis: A regression-based approach, 1, 12–20.

[ref33] Hayes A. F. (2017). Introduction to mediation, moderation, and conditional process analysis: A regression-based approach. New York: Guilford Press.

[ref34] Javed B. Naqvi S. M. M. R. Khan A. K. Arjoon S. Tayyeb H. H. (2019). Impact of inclusive leadership on innovative work behavior: the role of psychological safety. J. Manag. Organ. 25, 117–136. doi: 10.1017/jmo.2017.3

[ref35] Jolly P. M. Kong D. T. Kim K. Y. (2021). Social support at work: an integrative review. J. Organ. Behav. 42, 229–251. doi: 10.1002/job.2485

[ref36] Judge T. A. Piccolo R. F. Ilies R. (2004). The forgotten ones? The validity of consideration and initiating structure in leadership research. J. Appl. Psychol. 89, 36–51. doi: 10.1037/0021-9010.89.1.36 14769119

[ref37] Kim J. Y. Yoon D. Y. (2021). The effects of authentic leadership on adaptive performance: the moderated mediation effect of relational energy and promotion focus. Korean J. Manage. 29, 67–98. doi: 10.26856/kjom.2021.29.2.67

[ref38] Kinicki A. J. Latack J. C. (1990). Explication of the construct of coping with involuntary job loss. J. Vocat. Behav. 36, 339–360. doi: 10.1016/0001-8791(90)90036-2

[ref39] Kirkman B. L. Rosen B. (1997). A model of work team empowerment. Res. Organ. Chang. Dev. 10, 131–167.

[ref40] Kundu S. C. Kumar S. Gahlawat N. (2019). Empowering leadership and job performance: mediating role of psychological empowerment. Manag. Res. Rev. 42, 605–624. doi: 10.1108/MRR-04-2018-0183

[ref41] Lee S. Cheong M. Kim M. Yun S. (2017). Never too much? The curvilinear relationship between empowering leadership and task performance. Group Org. Manag. 42, 11–38. doi: 10.1177/1059601116646474

[ref42] LePine J. A. Colquitt J. A. Erez A. (2000). Adaptability to changing task contexts: effects of general cognitive ability, conscientiousness, and openness to experience. Pers. Psychol. 53, 563–593. doi: 10.1111/j.1744-6570.2000.tb00214.x

[ref43] London M. Mone E. M. (1999). Continuous learning. In Ilgen D. R. Pulakos D. E. (Eds.), The changing nature of performance: Implication for staffing, motivation, and development, San Francisco, CA: ossey-Bass. 119–153.

[ref44] Lorinkova N. M. Perry S. J. (2017). When is empowerment effective? The role of leader-leader exchange in empowering leadership, cynicism, and time theft. J. Manag. 43, 1631–1654. doi: 10.1177/0149206314560411

[ref45] MacCallum R. C. Browne M. W. Sugawara H. M. (1996). Power analysis and determination of sample size for covariance structure modeling. Psychol. Methods 1, 130–149. doi: 10.1037/1082-989X.1.2.130

[ref46] Moglia M. Hopkins J. Bardoel A. (2021). Telework, hybrid work and the united Nation’s sustainable development goals: towards policy coherence. Sustain. For. 13:9222. doi: 10.3390/su13169222

[ref47] Morgan P. J. Cleave-Hogg D. DeSousa S. Tarshis J. (2004). High-fidelity patient simulation: validation of performance checklists. Br. J. Anaesth. 92, 388–392. doi: 10.1093/bja/aeh081 14742327

[ref48] Morrison E. W. Phelps C. C. (1999). Taking charge at work: Extrarole efforts to initiate workplace change. Acad. Manag. J. 42, 403–419. doi: 10.2307/257011

[ref49] Mumford M. D. Baughman W. A. Threlfall K. V. Uhlman C. E. Costanza D. P. (1993). Personality, adaptability, and performance: performance on well-defined problem solving tasks. Hum. Perform. 6, 241–285. doi: 10.1207/s15327043hup0603_4

[ref50] Niessen C. Jimmieson N. L. (2016). Threat of resource loss: the role of self-regulation in adaptive task performance. J. Appl. Psychol. 101, 450–462. doi: 10.1037/apl0000049 26348477

[ref51] Nijssen M. Paauwe J. (2012). HRM in turbulent times: how to achieve organizational agility? Int. J. Hum. Resour. Manag. 23, 3315–3335. doi: 10.1080/09585192.2012.689160

[ref52] Nonaka I. (1991). The knowledge-creating company. Harvard business review. Available at: https://hbr.org/2007/07/the-knowledge-creating-company

[ref53] Nonaka I. Takeuchi H. (1996). The knowledge-creating company: how Japanese companies create the dynamics of innovation. Long Range Plan. 29:592. doi: 10.1016/0024-6301(96)81509-3

[ref54] Oliver S. Reddy Kandadi K. (2006). How to develop knowledge culture in organizations? A multiple case study of large distributed organizations. J. Knowl. Manag. 10, 6–24. doi: 10.1108/13673270610679336

[ref55] Peng H. (2013). Why and when do people hide knowledge? J. Knowl. Manag. 17, 398–415. doi: 10.1108/JKM-12-2012-0380

[ref56] Pitafi A. H. Liu H. Cai Z. (2018). Investigating the relationship between workplace conflict and employee agility: the role of enterprise social media. Telematics Inform. 35, 2157–2172. doi: 10.1016/j.tele.2018.08.001

[ref57] Plonka F. E. (1997). Developing a lean and agile work force. Human Factors and Ergonomics in Manufacturing 7, 11–20. doi: 10.1002/(SICI)1520-6564(199724)7:1<11::AID-HFM2>3.0.CO;2-J

[ref58] Ployhart R. E. Bliese P. D. (2006). “Individual adaptability (I-ADAPT) theory: conceptualizing the antecedents, consequences, and measurement of individual differences in adaptability” in Understanding adaptability: A prerequisite for effective performance within complex environments, vol. 6 (Emerald: Group Publishing Limited), 3–39.

[ref59] Podsakoff P. M. MacKenzie S. B. Lee J. Y. Podsakoff N. P. (2003). Common method biases in behavioral research: a critical review of the literature and recommended remedies. J. Appl. Psychol. 88, 879–903. doi: 10.1037/0021-9010.88.5.879 14516251

[ref60] Porter C. M. (2018). Long live social exchange theory. Ind. Organ. Psychol. 11, 498–504. doi: 10.1017/iop.2018.102

[ref61] Pulakos E. D. Arad S. Donovan M. A. Plamondon K. E. (2000). Adaptability in the workplace: development of a taxonomy of adaptive performance. J. Appl. Psychol. 85, 612–624. doi: 10.1037/0021-9010.85.4.612 10948805

[ref62] Rao Jada U. Mukhopadhyay S. Titiyal R. (2019). Empowering leadership and innovative work behavior: a moderated mediation examination. J. Knowl. Manag. 23, 915–930. doi: 10.1108/JKM-08-2018-0533

[ref63] Reychav I. Weisberg J. (2010). Bridging intention and behavior of knowledge sharing. J. Knowl. Manag. 14, 285–300. doi: 10.1108/13673271011032418

[ref64] Ryan R. M. Deci E. L. (2000). Intrinsic and extrinsic motivations: classic definitions and new directions. Contemp. Educ. Psychol. 25, 54–67. doi: 10.1006/ceps.1999.1020 10620381

[ref65] Salmen K. Festing M. (2022). Paving the way for progress in employee agility research: a systematic literature review and framework. Int. J. Hum. Resour. Manag. 33, 4386–4439.

[ref9001] Schmitt N. Cortina J. M. Ingerick M. J. Wiechmann D. (2003). Personnel selection and employee performance.

[ref66] Sharifi H. Zhang Z. (1999). A methodology for achieving agility in manufacturing organisations: an introduction. Int. J. Prod. Econ. 62, 7–22. doi: 10.1016/S0925-5273(98)00217-5

[ref67] Sherehiy B. Karwowski W. (2014). The relationship between work organization and workforce agility in small manufacturing enterprises. Int. J. Ind. Ergon. 44, 466–473. doi: 10.1016/j.ergon.2014.01.002

[ref68] Singh S. K. (2008). Role of leadership in knowledge management: a study. J. Knowl. Manag. 12, 3–15. doi: 10.1108/13673270810884219

[ref69] Spreitzer G. M. (1995). Psychological empowerment in the workplace: dimensions, measurement, and validation. Acad. Manag. J. 38, 1442–1465. doi: 10.2307/256865

[ref70] Srivastava A. Bartol K. M. Locke E. A. (2006). Empowering leadership in management teams: effects on knowledge sharing, efficacy, and performance. Acad. Manag. J. 49, 1239–1251. doi: 10.5465/amj.2006.23478718

[ref71] Troise C. Corvello V. Ghobadian A. O'Regan N. (2022). How can SMEs successfully navigate VUCA environment: the role of agility in the digital transformation era. Technol. Forecast. Soc. Chang. 174:121227. doi: 10.1016/j.techfore.2021.121227

[ref72] Tung H. L. (2014). When empowering leadership links to team work outcomes: encouraging the expression of psychological empowerment and knowledge sharing. GSTF J. Psychol. (JPsych) 1, 33–40. doi: 10.5176/2345-7872_1.2.17

[ref73] Van Den Hooff B. De Ridder J. A. (2004). Knowledge sharing in context: the influence of organizational commitment, communication climate and CMC use on knowledge sharing. J. Knowl. Manag. 8, 117–130. doi: 10.1108/13673270410567675

[ref74] Wang S. Noe R. A. (2010). Knowledge sharing: a review and directions for future research. Hum. Resour. Manag. Rev. 20, 115–131. doi: 10.1016/j.hrmr.2009.10.001

[ref75] Wang H. Sun J. (2019). The negative effects of empowering leadership: theoretical mechanisms and boundary conditions. Adv. Psychol. Sci. 27, 858–870. doi: 10.3724/SP.J.1042.2019.00858

[ref76] Xu Y. Zhang M. (2022). The study of the impact of empowering leadership on adaptive performance of faculties based on chain mediating. Front. Psychol. 13:938951. doi: 10.3389/fpsyg.2022.938951 35783713 PMC9247655

[ref77] Zhang X. Bartol K. M. (2010). Linking empowering leadership and employee creativity: the influence of psychological empowerment, intrinsic motivation, and creative process engagement. Acad. Manag. J. 53, 107–128. doi: 10.5465/amj.2010.48037118

[ref78] Zhang X. Qian J. Wang B. Chen M. (2020). The role of reward omission in empowering leadership and employee outcomes: a moderated mediation model. Hum. Resour. Manag. J. 30, 226–243. doi: 10.1111/1748-8583.12260

